# Model of the adaptive immune response system against HCV infection reveals potential immunomodulatory agents for combination therapy

**DOI:** 10.1038/s41598-018-27163-0

**Published:** 2018-06-11

**Authors:** Ayesha Obaid, Anam Naz, Aqsa Ikram, Faryal Mehwish Awan, Abida Raza, Jamil Ahmad, Amjad Ali

**Affiliations:** 1 Atta-ur-Rahman School of Applied Biosciences (ASAB), National University of Sciences and Technology (NUST), Islamabad, Pakistan; 2 National Institute of Lasers and Optronics (NILOP), Islamabad, Pakistan; 3 Research Center for Modeling and Simulation (RCMS), National University of Sciences and Technology (NUST), Islamabad, Pakistan

## Abstract

A regulated immune system employs multiple cell types, diverse variety of cytokines and interacting signalling networks against infections. Systems biology offers a promising solution to model and simulate such large populations of interacting components of immune systems holistically. This study focuses on the distinct components of the adaptive immune system and analysis, both individually and in association with HCV infection. The effective and failed adaptive immune response models have been developed followed by interventions/perturbations of various treatment strategies to get better assessment of the treatment responses under varying stimuli. Based on the model predictions, the NK cells, T regulatory cells, IL-10, IL-21, IL-12, IL-2 entities are found to be the most critical determinants of treatment response. The proposed potential immunomodulatory therapeutic interventions include IL-21 treatment, blocking of inhibitory receptors on T-cells and exogenous anti-IL-10 antibody treatment. The relative results showed that these interventions have differential effect on the expression levels of cellular and cytokines entities of the immune response. Notably, IL-21 enhances the expression of NK cells, Cytotoxic T lymphocytes and CD4+ T cells and hence restore the host immune potential. The models presented here provide a starting point for cost-effective analysis and more comprehensive modeling of biological phenomenon.

## Introduction

Around 71 million individuals are infected chronically with Hepatitis C Virus (HCV) worldwide, with a greater risk of liver cirrhosis and hepatic tumours^1^. HCV eradication around the globe is still a long way off. One of the reasons due to which HCV infection flourishes chronically is the inability of the host immune system to develop effective antiviral immune response^2^. In fact, various molecular or protein interactions within innate or adaptive immune signalling pathways are directly associated to the HCV infection (either chronic infection or virus elimination)^3,4^. Besides this, HCV has evolved potential approaches to defend against host immune system, at various levels^2^, which results in a persistent battle between the multifaceted immunogenic host response and HCV for the control of the host machinery. As a result, either host clears the infection or the viral proteins take over the host machinery and replicate indefinitely. Efficacious innate as well as adaptive immune responses are vital in the clearance of the virus. There are multiple integrating immune partners executing a coordinated effort to produce an immune response against HCV^4,5^. Furthermore, the immune response to HCV infection is governed by several cytokines (activating/deactivating) and whose balance is critical for the immune modulatory activities occurring in the liver^2,6^. Yet, the functional role of different cell and their subtypes producing similar cytokines under various alternating stimuli, remains elusive^7^. The immune system detects such key factors and then translates them into effector functions at various levels employing specialized immune cells such as dendritic cells (DCs), natural killer (NK) cells, CD4+ and CD8+ T cells, B cells and macrophages^7^. Alternatively, the failure of adaptive immune responses against the viral infection is mainly because of evolving viral escape strategies which includes mutations and changes in the effector functions^2^.

Up till now, several studies have proposed the probable mechanisms leading towards the failure of host adaptive immune response. However, it is yet hard enough to extricate the exact causes and consequences of viral persistence. We believe a holistic model of the biological adaptive immune signalling mechanism is essential for deciphering the HCV disease pathology and designing alternative and possibly new multi-drug therapies. However, the plethora of signalling pathways involved in HCV infection comprise a multifaceted dynamical system whose complexity and wide interacting network makes it difficult to study via conventional experimentation approaches. Similarly, there are limitations in the existing methodologies as they can only interpret limited number of proteins and their interactions with other proteins and immunomodulatory agents and thus may not be able to cover the whole system, at a time. Systems biology approaches offers good alternative to existing strategies to model and analyse large networks^8,9^. Mechanistic hypotheses related to biological problems could easily be tested by applying appropriate mathematical models. In this context, several mathematical models have been employed successfully to analyse and investigate the integrated signalling networks and dynamic behaviours of the entities (Genes, RNAs and Proteins) involved^10,11^.

Biological systems are modelled using several mathematical frameworks including stochastic or differential equations (PDEs, ODEs, PLDEs, DDEs) or networks based on graph theory (Logical, Boolean, Bayesian)^12^. Usually, biological networks remain highly complex and dynamic in nature and there is no experimental data available for all the entities, such as enzyme kinetics or logical parameters to manually construct ODE models of larger networks. Moreover, it also remains a challenge for these mathematical models to handle large networks which are subjected to state space explosion phenomenon^13^. Therefore, alternative approaches can be adopted which can approximately model the dynamic behaviours of biological systems and get insights into the signal flow within associated biological networks^11^. One such approach is the use of Petri nets (PN) theory^14^. PNs are based on graph theory and have the potential to model different types of frameworks including biochemical processes, chemical reactions, biological networks (cellular or molecular), industrial models, etc., with flexible and simple representation^14^. PN models are usually used to describe generic principles and can be applied on abstracted models efficiently. The ease of modelling and interpretation makes PN theory suitable approach to model large networks where kinetic knowledge is lagging for few or most of the entities involved. PN approach used in the study emphasizes the structure of biological signalling pathway, as it is believed that the molecular interactions within a network have evolved to such an extent that they have a stabilizing effect on the signalling network. Thus it can be assumed that the network connections are foremost and critical factor for signal propagation through the signalling pathway^11,15^.

We have employed the computational systems biology methods coupled with mathematical modelling to comprehensively analyse the integrated HCV-induced adaptive immune signalling pathway. The unavailability of a comprehensive mathematical model explaining the adaptive immune responses against HCV led us to design a PN model, that enabled us to characterize the host immunogenic entities playing a significant role in the clearance of viral infection. Since the selected network is quite complex with large number of entities thus PN modelling is considered the best suited approach. The proposed model represents the wide-ranging HCV-induced adaptive immune system while preserving the behaviours of the signalling proteins, previously established experimentally. The model is able to perceive those properties and behaviours which are not apparently evident in the individual experimental studies. The models enhanced our understanding of the phenomenon of adaptive immune response system during HCV infection. Once the model was verified, few of the perturbation experiments were applied to propose immunomodulatory therapies to augment the existing IFN-α/RBV therapy. The ease of *in silico* experimentations provided in PNs model could assist in the development of new immunomodulatory treatment regimens along with the discovery therapeutic vaccines against chronic HCV and other viral infections. The modelling approach applied in this study is straightforward and can be extended to other biological systems to better explore specific behaviours.

## Results

### Logic based diagram depicting the essential features of the adaptive immune signalling during HCV infection

**Figure 1:**
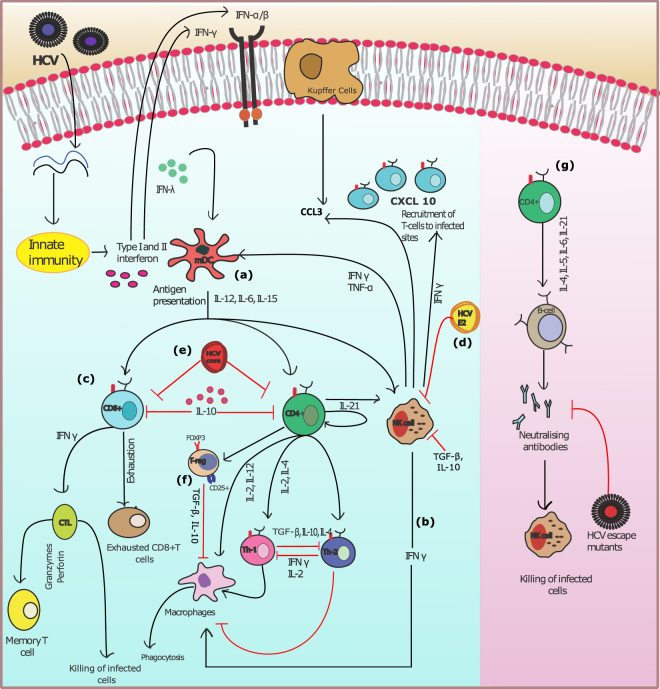
HCV infection and key adaptive immune responses: As soon as HCV RNA is recognized by host cells in the liver (Hepatocytes). In response, innate immune system produces Type I and II interferons to trigger an antiviral state^16,18^. While adaptive immune response is initiated by several main entities in the immune system. They include, (**a**) memory Dendritic cells (mDC) that activate CD8+ cells, CD4+ cells and natural killer (NK) cells by releasing cytokines IL-15, IL-4, IL-12^18^ (**b**) NK cells produce interferon-γ to mediate antiviral effects. (**c**) CD8+ cells and CD4+ cells control the T- helper cells (Th1 & Th 2) which as a result regulate the macrophages functionality, induce Cytotoxic T cells (CTLs) and T-regulatory cells (Tregs)^7^ (**d**) CD81 NK receptor is blocked by HCV E2 protein, reducing release of interferon-γ and cytotoxic particles by NK cells^23^ (**e**) MHC class I expression is increased on affected hepatocytes by HCV core protein, hence reducing activity of NK cell against affected cells^88^. HCV also rises the regulatory T cells in liver^24^. (**f**) NK cells activity is reduced by regulatory T cell secreting IL-10 and transforming growth factor–β (TGF -β)^24^. (**g**) Humoral responses are activated via CD4+ cells by the release of IL-21, IL-4, IL-6 and IL-5.

The logic based diagram of adaptive immune response (Fig. 1) is a comprehensive and detailed visual representation of the complex molecular regulation, orchestrated by series of signalling pathways based on experimental data. This illustration highlights the complexity of the model and the extent of interactions amongst the diverse cell populations. As demonstrated in Fig. 1, significant signalling cascades triggered in adaptive response by the activation of interferons, several immune related cytokines, DCs, NK cells, CD4+ and CD8+ T cells, B-cells which cooperate to defend hepatic cells against HCV infection. These signalling cascades are generally observed and studied as distinct entities instead of an integrated complex of molecular interactions. Thus, in order to study the system holistically, a comprehensive network is formulated by assimilating significant interactions recognized in various earlier experimental studies. The arrangement of the components/entities and their interactions in the designed pathway facilitate the visualization of paths and events followed by the system from the preliminary cause to the ultimate outcome.

Typically, the immune signalling pathways are triggered by the HCV particle which targets the hepatocytes and releasing its RNA. The primary line of defence is the generalized innate immune response against the viral infection which afterwards activates more specified adaptive immune response elements^16^. The intercellular signalling (systematized by chemokine, cytokines and cell surface receptors) and intracellular signalling (achieved by the network of signalling pathways) is induced through several vital regulatory immune elements^17^. Once the HCV RNA is released, the type I and III interferons are produced through innate response^4^. Subsequently, it stimulates the adaptive responses via activating the NK cells and DCs residing in the liver^18^. DCs are crucial for recognizing pathogens in addition to the triggering of adaptive immunity. Then they further activate NK cells (reciprocal activation), T-cells (CD4+ cells, CD8+ cells and T-regulatory cells, Cytotoxic T lymphocytes (CTLs)), by releasing interferon γ, λ along with several other cytokines as shown in the Fig. 1^18,19^.

### Crosstalk between innate and adaptive immunity mediated by NK cells

Initially, NK cells establish host innate defence to counter viral infections. In case of HCV infection, NK cells are triggered through type IFN-I (α, β) produced by affected hepatocytes^20^. In addition to it, NK cells can also be activated via IL-12 secreted by DCs, hence sanctioned to eradicate infected cells^21^. Although classically NK cells are viewed as components of innate immune system, but it has been shown clearly in several studies that they have a substantial part to play during adaptive immune response as well^22^. They are a major source of IFN-γ and tumour necrosis factor-alpha (TNF-α) which hinder viral replication without destroying the hepatocytes^22^. Moreover, they may bring about partial or complete DCs maturation. NK cell activity is rigorously directed by the stimulating and inhibiting NK receptors (NKRs) which mainly consist of inhibitory killer Ig–like receptors (KIRs)^20–24^. Furthermore, NK cells also exhibit reciprocal and regulatory interactions with DCs, macrophages, T and B cells thus operating to intensify or diminish immune response reactions^25,26^.

### Virus-specific T-cell mediated responses

T-cells specific antiviral response is the main component of the immune system to control viremia^7^. Many T cell subsets have been characterized depending upon the expression of distinct cell surface markers and/or the effector molecules produced by a particular T-cell population. HCV-specific CD8+ T cells are able to control virus by two effector approaches (a) they can cytotoxically destroy affected target cells (mainly liver cells) that use HLA class I molecules to present viral antigens on their surface and (b) they also play a role in controlling viruses by non-cytolytic mechanisms, including cytokines secretion (such as TNF-α, IFN-γ)^2,3,5,16,17,20,27^. Helper CD4+ T cells support the functional mechanisms of cytotoxic CD8+ T cells^28^. Along with that, the initiation of co-stimulatory signalling pathways facilitate the cytotoxic potential by the release of cytokines including IFN–γ and IL-2^29^. Various regulatory T cells (Tregs) also participate in HCV immunology. Tregs have been associated to HCV-specific exhausted T cell phenotype during early infection phase, causes failure of T-cell and chronic infection. Tregs act as a shield from immunopathological effects by chronic inflammation^7^. Induction of Tregs can be enhanced by elevated levels of TGF-β, mainly produced by affected liver cells^24,30^.

### Humoral immune responses during HCV infection

Majority of the patients chronically infected with HCV have HCV-specific neutralizing antibodies but they are limited in functionality. Several mechanisms are involved in evasion of viral particles from humoral immune response^2,4,16^. Hence, viral quasispecies evolution that exhibit alterations within target epitopes leading to viral escape from neutralizing antibodies^31^. Thus, their ability to control and clear the infection is limited in case of HCV.

### Adaptive immune response failure in controlling HCV

Several mechanisms that exhibit an HCV-specific anomaly towards immunity have been stated in previous studies^2,16,27^. Amongst them, failure of sustained antiviral responses by T cell is major determinant of persistent/chronic HCV infection. The important mechanisms in T-cell response failure include (a) incapability of effector T cells to migrate towards the site of infection in liver as well reduced antigenic presentation (b) another contributing mechanism to T cell dysfunction include expression of inhibitory receptors. The key factors in failure of immune process include constant antigen activation, reduced help of CD4+ T cell along with the activation of Tregs^2,3,27^. T cell responses (CD4+ and CD8+) specifically against HCV are noticeable in persistent infection but ineffective against HCV. Nevertheless, CD4+ and CD8+ T cells obtained from HCV infected chronic patients exhibit maturation anomalies consisting of decreased cytotoxic capacities, lesser Th1-type cytokines secretion as well as a minimum proliferative potential as a result of *ex vivo* antigenic stimulation^16^. T-helper cells prompt DCs to prime CD8+ T cells, identify antigens by CD8+ T, CD4+ T cells on the same APC (antigen-presenting cell) is probably the major characteristic of antigen-specific T cell help. Therefore CD4+ T cells failure may restrict chances of CD8+ T cells priming via completely stimulated HCV antigen- loaded DC^2,3,27^.

### Model based insights into the adaptive immune system during HCV infection

Several studies demonstrate that the consequences of a viral infection are governed by the host potential to elicit strong antiviral responses as well as viral preventive mechanisms^2–4,7,16,17,20,21,32^. The current study attempts to model these diverse mechanisms and explores the critical balance and limiting factors associated with the elimination of the virus. The HCV adaptive immune response model comprises of almost all the known players of the adaptive immune system. The cells include mainly CD4+ and CD8+ T cells, NK cells, macrophages, Tregs, Exhausted T cells, B-cells, antibodies and various cytokines (IFN-y, TNF-α, TGF-β, IL-10, IL-12, IL-21, IL-15, IL-2) mediating the cellular signalling (Fig. 1). All of these key players have been seen to be involved in viral clearance as reported in various studies^3,5,27^. Therefore, it is quite plausible to have a well-synchronized interaction of diverse kind of immune cells to estimate an effective immune response against HCV, nevertheless, very limited information about the exact interaction amongst these cross-talks are available.

We have attempted to generate PN models taking the knowledge based logic diagram (Fig. 1) into account, depicting various states during HCV infection. First, a baseline model (Supplementary File 1) was constructed exhibiting the basal levels of all the entities in the absence of any kind of infection. We refer to it as *Baseline Model*. The *Baseline model* efficiently demonstrates the normal behaviours of a control system. It was then extrapolated to include the effects of HCV infection and its proteins (Core, E2) on the system which resulted into two additional models. One representing the successful immune response leading towards clearance of infection, referred to as *Effective Adaptive Immune Response Model*, while the second model signifies that the system is moving towards chronicity and persistence of infection, referred to as *Failed Adaptive Immune Response Model*. Subsequently, a treatment response model was created to analyse the effect of IFN-α/RBV therapy on the immune system. Later, the predictive ability of the PN models was used to examine various immunomodulatory conditions and to predict and propose the best immunomodulatory therapeutic possibilities.

The model assumes that once acute infection is established, host machinery reacts to eliminate the virus by triggering immune responses. Alongside, the virus continues propagation by utilizing the available nucleotides and amino acids for replication and translation within the host. Viral and host proteins/cells and subtypes in the model are represented by continuous places, whose input transitions continuously add tokens to them with time. The immune cells and various subtypes, based on the expression of markers, are given a separate “place” in the model in order to easily differentiate and study the behaviour. Initially a token is transferred to each place by input transitions, which represents the existence of a protein and does not ascribe to the definite expression level of a protein. Furthermore, the processes occurring in the cells are represented by transitions. Here, the groupings of input and output edges (arcs) are dependent entirely upon the kind of cellular mechanisms and molecular interactions involved within. Thus, input and output edges are used in combinations to model various biological mechanisms that facilitate the flow of cellular signals. Time units are denoted by specific time blocks, in which each transition fires once. The mass action kinetics (1) is used to fire the transitions and the signal flow is primarily based on the interconnecting arcs of the network.

To verify the correctness of the model, simulations run was used to get relative expression values for all the cellular and cytokine responses. These values were then compared with the *Baseline model* (supplementary file 1) to get insights into the differential system behaviours during each state. These results emphasize on the multifaceted dynamics which triggers the adaptive immune response against HCV and also specifies the variable levels of biological implications at several stages of infection. For clarity in PN illustrations, cytokines are highlighted by green colour while immune cells are represented by blue colour.

### Model of the *Effective Adaptive Immune Response system*: NK cells mediators of innate and adaptive immune response crosstalk

Cytokine dynamics is thought to be the main regulatory factor in the entire immune system^6,33,34^. Cytokines have generally been divided into various categories depending upon its function, (a) pro-inflammatory cytokines including TNF-α and IL-6 (b)Th1 cytokines (IL-2, IL-12, IFN-γ), that are produced by Th1 activated lymphocytes, (c) Th2-type cytokines (IL-5, IL-4, IL-10). Th2 cytokines play a significant role in the downregulation of Th1 response, this leads to the inhibition of antigen-presentation on macrophage and promotes B-cell proliferation, resulting in specific antibody release.

**Figure 2:**
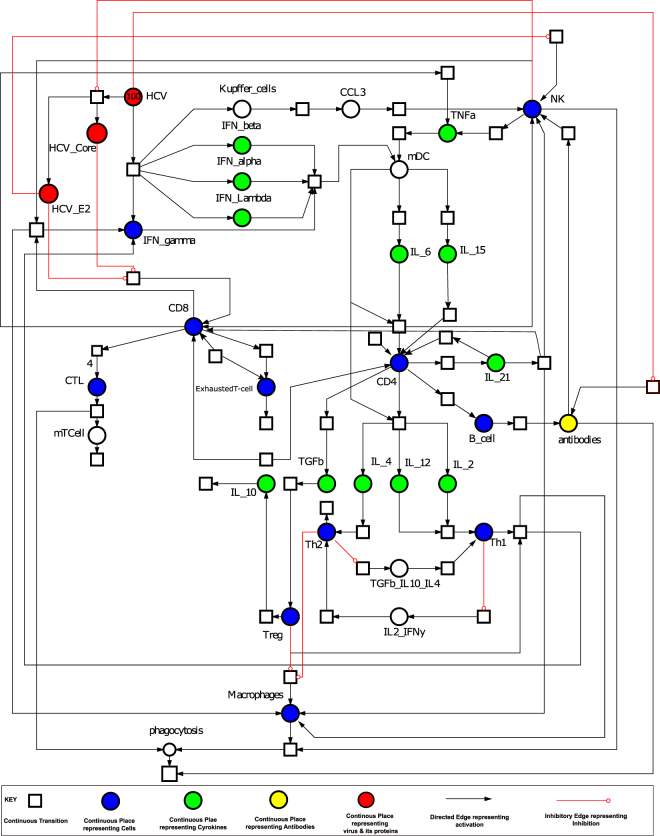
Illustration of the PN model representing *Effective Adaptive Immune Response* against HCV. A circle represents a continuous place, signifying HCV proteins, host signalling proteins, receptor complexes and cellular enzymes. A square box represents continuous transition, demonstrating all cellular mechanisms comprising of replication, transcription, translation, activation, deactivation, endocytosis and exocytosis. A directed arc links a standard transition to a standard place and vice versa. An inhibitory edge denotes the inhibitory outcome on a biological process by an entity (Enzymes, host and viral proteins). Arcs weights are equal to 1 except stated otherwise. Green coloured circles represent important cytokines, blue coloured circles represent cells involved in immune response, while red coloured circles represent HCV and its proteins.

**Figure 3:**
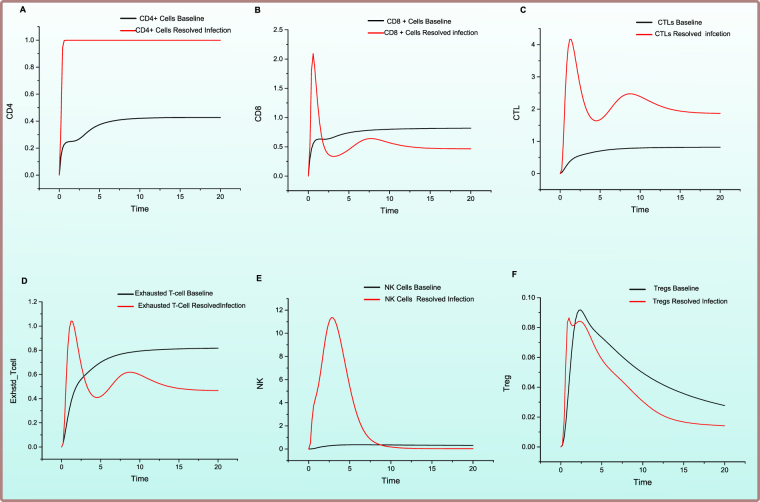
Comparison of relative change in expression levels of cellular entities during HCV infection and Effective Adaptive Immune Response. Y-axis represents the relative expression level of cellular entities during in *Effective Adaptive Response Model* as compared with *baseline model*, while X-axis shows time units. Black line signifies the relative activity level prior to HCV infection while red line represents the relative activity level afterward successful response to HCV.

**Figure 4:**
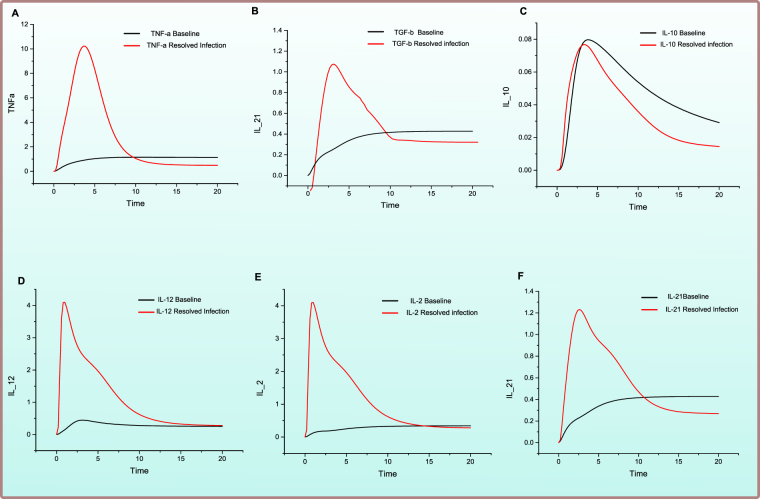
Comparison of relative change in expression levels of cytokines response during HCV infection and *Effective Adaptive Immune Response*. Y-axis represents the relative expression level of cytokines during in *Effective Adaptive Response Model* as compared with *baseline model*, while X-axis shows time units. Black line signifies the relative activity level prior to HCV infection while red line represents the relative activity level afterward successful response to HCV.

PN model of the *Effective Adaptive Immune Response* is illustrated in Fig. 2. The behaviour of significant entities and plausible relationship amongst various cytokines concentration is observed via simulations of the model and have been represented in Figs 3 and 4. These and many other biological observations are reproduced efficiently by the models generated in this study which verify the correctness and soundness of the models.

As observed in the model simulations of cellular response (Fig. 3), there is a surge in HCV CD4+ response (Fig. 3A) which helps in the increase of percentage and functional effects of CD8+ T cells (Fig. 3B). HCV CD4+ T cells are vital to adaptive response as they trigger both cytotoxic and humoral responses^35^. They are able to produce Th1- cytokines like IFN-γ that aids in the recruitment of neutrophils and macrophages and cause a robust inflammatory response. A multi-specific, robust, continuous, CD4+ -T-cell-specific Th1 response during HCV infection results in the clearance of infection. CTLs being the key effector cells, facilitate viral clearance using apoptosis-related cytolytic mechanisms by releasing type I cytokines; IFN-γ and TNF-α. The model clearly depicts that CTLs are substantially activated (Fig. 3C), leading to strong cytotoxicity and have the ability to produce strong IFN-γ response. The relative effect of helper CD4+ cells in spontaneous clearance of acute HCV infection was observed by Smyk-Pearson *et al*. and established the although HCV-specific CTLs are present during infection, and they are able to produce IFN – γ, proliferate, have cytotoxic behaviour but they still did not certify resolution of infection, but either these CTLs are initially primed with CD4+ T cell help or not was a vital factor^36^. An initial increase in exhausted T cells (Fig. 3D) is observed but later on the relative expression tappers down as the system moves towards resolution of infection. Amongst other cellular responses, a noticeable increase in NK cells (Fig. 3E) is detected during early infection. NK cells employ their antiviral activity via direct, non-MHC-restricted cytotoxic processes and production of IFN-γ^21^. NK cells also regulate other adaptive immune mechanisms either directly or indirectly. The crosstalk of NK cells particularly with DCs macrophages and T cells is important^21^. NK cells seem the chief cellular player mediating a crosstalk amongst innate and adaptive immune components. A plausible role of NK cells during HCV immune biology is also reinforced by the fact that they are stimulated during acute infection phase, as shown by an amplified expression of subsets NK cells^37^. This is contemporary to strong induction of IFN-γ and associated cytotoxicity. NK cells are crucial to the resolution of infection because NK cell lines that have been stimulated by cytokines and primary NK cells obtained from naive individuals and might lead to the lysis of HCV-replicating cells, predominantly at elevated effector-to-target ratios^38^ also, simultaneously secreting IFN-γ which inhibits HCV replication^39^. A small amount of Tregs (Fig. 3F) is also observed to be expressed during adaptive immune response. Although Tregs are negative regulators of T cells and promote the infection towards chronicity but their regulatory effect is required to protect the liver from damaging effects of inflammation^40^. The inhibitory effect is negligible here in these circumstances.

The notable cytokines whose variations strongly affect the outcome of infection are shown in Fig. 4. Among cytokine responses, IFN-γ is the main mediator of adaptive immunity, sets in motion various cellular responses and seems to be highly expressed as a response to infection. However, it was noted that the higher amounts of IFN-γ do not ensure recovery alone. Thus, in all the models’ simulations a very high amount of IFN-γ is present. A surge in the amount of TNF-α (Fig. 4A) signifies clearance of infection as it is an antiviral cytokine which effect the viral clearance as well as limiting the tissue damage. TGF-β (Fig. 4B) promotes infection related tumour development and tissue injury. In the resolved infection, comparatively lesser amount of TGF-β is observed. Importantly, the ratio of TGF-β and TNF-α is important rather than absolute standalone quantities. In resolved infection higher TNF-α to TGF-β is present (Fig. 4). As Tregs are activated in a small number thus, thus IL-10 (Fig. 4C) is also noted to be low as compared to chronic infection. IL-12 (Fig. 4D) is highly important cytokine regulating CD8+ cells and sanctioning their differentiation. On the other hand, IL-2 (Fig. 4E) is important for CD4+ mediated immune response and survival of CD8+ cells. The increase in the levels of IL-21 (Fig. 4F) is noted which is also a critical determinant in CD4+ T-cell response^41^. It helps in proliferation of NK cells and also mediating crosstalk amongst B-cells and T cells leading towards humoral responses. Thus, it induces both an adequate isotype switching in B-cells and an ADCC specialized NK cell subset response (Fig. 1)^41,42^. Main effects of successful adaptive immune response include but not limited to, increase in cytotoxicity via CTLs, increase in TNF-α and IFN-γ production, lower level of Tregs and IL-10. These effects have been reciprocated by our model making the model a good base to experiment further.

### Model of the *Failed Adaptive Immune Response*: Persistence of viremia leading towards chronic infection

**Figure 5:**
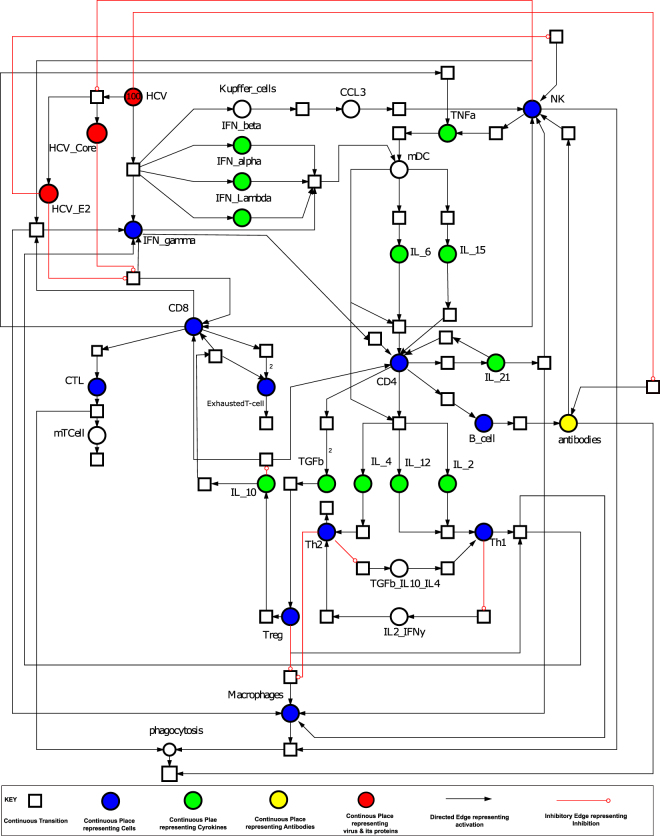
Illustration of the PN model representing *Failed Adaptive Immune Response* against HCV infection. A circle represents a continuous place, signifying HCV proteins, host signalling proteins, receptor complexes and cellular enzymes. A square box represents continuous transition, demonstrating all cellular mechanisms comprising of replication, transcription, translation, activation, deactivation, endocytosis and exocytosis. A directed arc links a standard transition to a standard place and vice versa. An inhibitory edge denotes the inhibitory outcome on a biological process by an entity (Enzymes, host and viral proteins). Arcs weights are equal to 1 except stated otherwise. Green coloured circles represent important cytokines, blue coloured circles represent cells involved in immune response, while red coloured circles represent HCV and its proteins.

**Figure 6:**
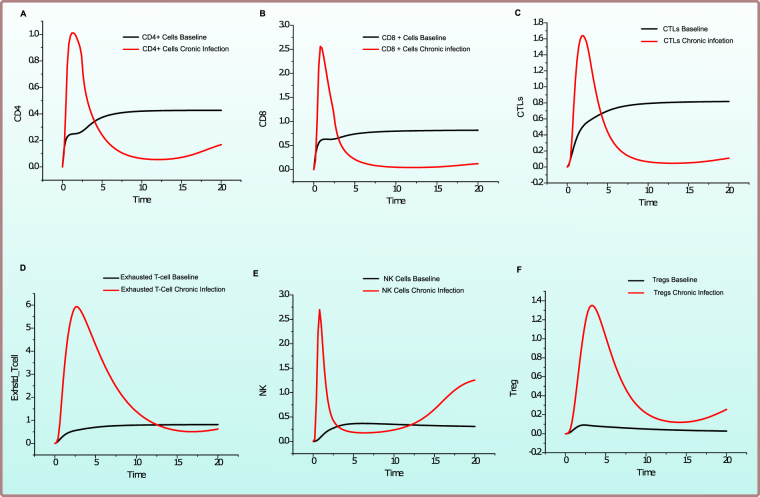
Comparison of relative change in expression levels of cellular response during HCV infection and *Failed Adaptive Immune Response*. Y-axis represents the relative expression level of cellular entities during in *Failed Adaptive Response Model* as compared with *baseline model*, while X-axis shows time units. Black line signifies the relative levesl prior to HCV infection while red line represents the relative levesl afterward failure of immune response to HCV.

**Figure 7:**
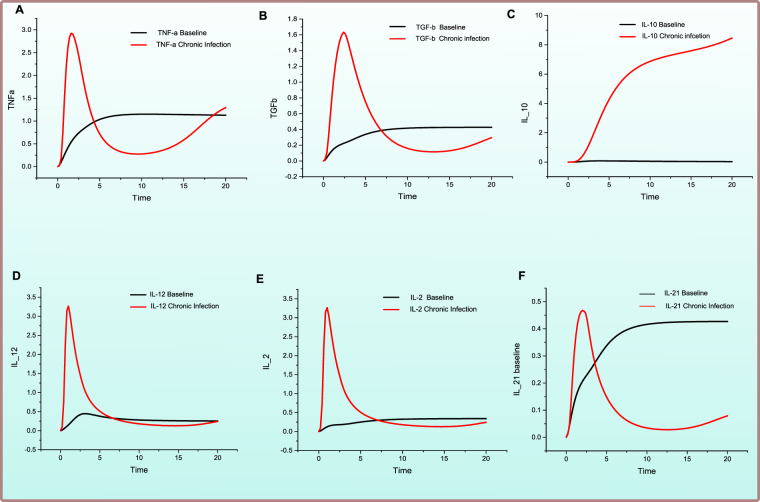
Comparison of relative change in activity levels of cytokine responses to chronic HCV infection and *Failed Adaptive Immune Response*. X-axis depicts time units while the Y-axis signifies relative activity level variation in the cytokine entities during failed adaptive immune response when compared with *baseline model* PN (Supplementary File 1). Black line shows the relative activity level before any sort of HCV infection is present while red line shows the relative activity level after establishment of chronic HCV infcetion.

In case of failed host immune response, it leads towards the persistence of viremia and chronic infection. The key regulators in such a scenario have been modelled and are represented in Figs 5, 6 and 7. The PN is illustrated in Fig. 5 while simulation results are shown in Figs 6 and 7 comparing the relative concentration levels of cellular and cytokine responses (in case of failed immune response) with baseline model (Supplementary File 1).

It is observed in Fig. 6, in the absence of viral clearance, there is a decrease in CD4+ cellular response (Fig. 6A) as compared to a resolved infection (Fig. 3A). This results in scarce production of Th1-type cytokines along with decreased proliferation in response to stimulation by an antigen. CTLs are lower in concentration (Fig. 6C) and with impaired proliferation and cytokine production as a result of comparatively low IFN-γ production. Evidence also suggests that during chronic HCV infection, HCV specified CTLs are few exhibiting decreased performance, they also display anergic features with reduced inducement of type I IFNs^6^. The CD8+ T cells experience exhaustion phenomenon (Fig. 6D) due to chronic antigenic stimulation leading to the increased expression of inhibitory receptors such as Programmed cell death protein 1 (PD-1), Cytotoxic T-lymphocyte-associated protein 4 (CTLA4) and T cell immunoglobulin and mucin domain 3 (TIM3) receptors. T-cell exhaustion is believed to be a critical determinant during chronic infection leading towards reduced cytotoxicity and persistence of infection^43^. Convincing evidences are present for exhaustion and anergic T cells during HCV chronic infection^44,45^. Elevated levels of PD-1, TIM3 and CTLA4 expression are related to CTL dysfunction, resulting in lesser quantities of TNF-α and IFN-γ, as compared to their counterparts^44,45^. The core protein of HCV also disrupts host adaptive immune response by effecting the expression of PD-L1, which supports T cell dysfunction. Thus, leading towards lower CD8+ cell functionality. NK cells count lowers (Fig. 6E) as a result of HCV E2 protein inhibiting the function of NK cells by crosslinking CD 81receptor^46^. Furthermore, a relatively high level of Tregs (Fig. 6F) is observed during chronic infection which downregulates the effecter function of immune cells.

The analysis of cytokines response revealed that TNF-α to TGF-β ratio is low as compared to resolved infection. It is observed that TNF-α (Fig. 7A) is relatively reduced while TGF-β (Fig. 7B) production is increased. IL-10 (Fig. 7C) is highly increased which strongly attenuate the proliferation of CD8+ T cells and CD4+ T cells. TGF-β being a regulator of thymic T–cell development and differentiation, maintains T-cell homeostasis^47^. These immunomodulatory cytokines are believed to be negative repressors of inflammation and regulate hepatic immunity. Alternative probable mechanism of aberrantly regulated cytokines in chronic infection results from Tregs mediated immune regulation. These cells release both IL-10 and TGF-β in high amount thus inhibiting proliferation as well as cytokine release via T cells, either directly or by other related mechanisms^48^. Suppression of IL-12 (Fig. 7D) and IL-2 (Fig. 7E) occurs during chronic infection leading to low production of IFN-γ via T cells. Also, it leads towards more Th-2 mediated mechanisms. IL-21 expression is also lowered (Fig. 7F) which might lead to impaired humoral responses. IL-21 is negatively correlated with HCV RNA^49^ thus IL-21 producing CD4+ cells might help in the rescue of HCV specified CD8+ T cells for the control of viremia.

The main effects of failure of immune response are characterized by inhibition of NK cells and T cells by HCV proteins (core, E2), lower cytotoxicity by CTLs and CD8+ cell dysfunction leading to exhausted T cells response.

### Treatment response model

Once it is established that the model satisfies all the major features of various stages of infection, we further explored how the system supposed to behave on introduction of any treatment into the system. Hence, the current immunomodulatory treatment of HCV, which comprises of PEGylated interferon-α (IFN-α) and ribavirin (RBV), has been employed in the model. As PegIFN-α/RBV have been regarded as standard of care (SOC) for a long time, with sustained virological response (SVR) rate estimated at 40–50% in patients infected with genotypes 1 and 4 and up to 80% SVR rates in individuals with genotypes 2 and 3^50^. Where SVR is defined as the HCV RNA that is undetectable after completion of treatment and even after six months^50^. Although direct antiviral agents have shown a promising result in terms of lowering the viral infection but these treatments are reportedly hindered by resistance and relapse. The prime goal of DAAs is to inhibit the specific viral proteins which helps in its replication. Contrary to it, the immunomodulatory treatments enhance the hosts own immune response mechanisms to eliminate the virus from the body. Therefore, the immunomodulatory treatments cannot be ruled out of HCV treatment regimens.

**Figure 8:**
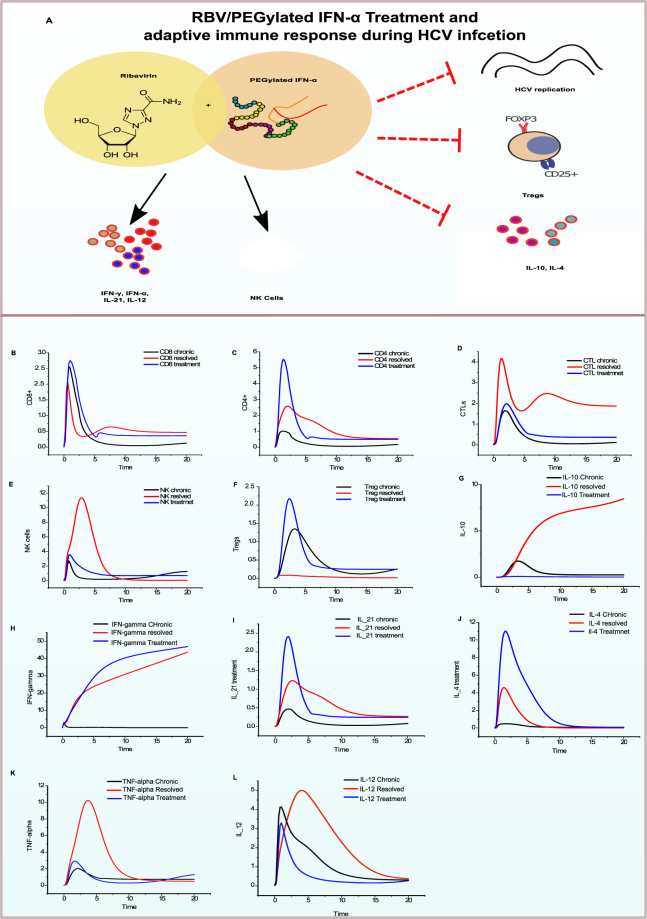
Adaptive immune response during treatment (PEGylated IFN-α/RBV). (**A**) Represents probable effects of PegIFN-α and RBV on adaptive responses during treatment. The treatment model was simulated and the resulting graphs are presented. x-axis represents time while y-axis depicts relative concentration levels. Black line represents the relative expression levels of the entity during chronic infection, red line represents the activity levels during resolved infection, while blue line represents the change in activity levels after treatment is introduced in the system.

Consequently, we extended the model by introducing the entities such as PEGylated-IFN-α and RBV to explore the performance of various cellular and cytokine responses during treatment stage (Fig. 8). Accordingly, a *Treatment response model* was generated and verified that it can accurately recapitulate the basic mechanisms and adaptive responses during IFN-α/RBV treatment of chronic HCV infection. The effects observed during the treatment response model are analysed according to the mechanism of action of both agents mentioned in literature.

Several theories regarding the mechanism of action of ribavirin have been developed. RBV is known to be a guanosine analogue supposed to elevate the effector function of IFN^51^ and avert relapse by raising the mutation rate of HCV^52^. RBV is also known to deplete the intracellular guanosine triphosphate (GTP) reservoir by acting as an inhibitor of inosine mono-phosphate dehydrogenase (IMPDH)^53^. HCV RNA–dependent RNA polymerase is directly affected by antiviral RBV^51–55^. Moreover, RBV greatly effects the adaptive immune responses as it is involved in tipping the T helper balance from a Th2 cytokine profile to an effective antiviral Th1 cytokine profile (Fig. 8).

Besides, IFN-α therapy induces high rates of SVR by preservation of substantial multi-specific HCV-specific CD4+ T cell responses (Fig. 8C)^56^. It is predicted that the treatment restores the IFN-γ production (Fig. 8H) via NK cells as they induce cytotoxicity that is related to virologic response^57^. Therefore, NK cell activation specifies responsiveness to IFN-α–based treatment and proposes the connection of the innate immune cells to viral clearance. However, in the model the there is no significant elevation in the NK cells levels which might explain the treatment failure or reduced response to treatment. IFN-α has several effects in stimulating the immune system. However, some mechanisms of IFN-α could perhaps have negligible effects or more significant ones as treatment lingers on.

It was observed through the model simulations that HCV replication is halted to downregulate the production of HCV proteins. However, we are interested to demonstrate the important cytokine and cellular regulators of adaptive immunity which are differentially affected during the course of HCV treatment. Several cellular responses and cytokines levels altered during treatment perturbations introduced in the model. Amongst them, a noteworthy rise in CD4+ cells (8 C), cytokine such as IL-21 (8I) and IL-4 (8 J) and a strong reduction in IL-10 is observed (8 G). It is also known that higher IL-10 concentration levels are present in the chronic infection^58–60^. After therapy response, the IL-10 levels were observed to be downregulated (Fig. 8G). Moreover, the IL-12 levels are found low in response to treatment as compared to chronic infection (Fig. 8L). However, high expression levels of IL-12 are determinants of resolved infection^33,55^. Both IFN-α and RBV differentially regulate the Th1 and Th2 cytokines as shown in several studies^51,55,61^. Their combined effects result in the suppression of IL-10 production but maintains a relatively good expression levels of IL-12, this eventually favours effector T cells for viral clearance. However, it is not always the case during the patients’ treatment response. That is why this balance can be toppled very easily by various other related partners involved which might result in the failure of the treatment. In the case of treatment failure, the chronic increase in PD-1 and other inhibitory receptors expression on T lymphocytes is observed, and hence an increase in exhausted T cells and Tregs. The most critical factors highlighted during the analysis of the *Treatment response model* include IL-10, IL-21, IL-12, IL-2 which determine the treatment outcome in terms of clearance of infection by modulating pro-inflammatory response. These determinants were selected for further analysis to propose combination therapy of immunomodulatory agents in conjunctions with PEGylated-IFNα/RBV treatment.

In summary, IFN-α/RBV acts to enhance the immune system and regulate the negative effects of immune activation. The discrepancies observed in responders and non-responder patients seem to be mediated by the inherent defects or differences in baseline activation of several cytokines in various individuals. It is primarily based on the immune signatures unique to every individual. It is noteworthy that the microenvironment of each cell is different and it might affect the cytokine balance very precisely. Pinpointing those limiting factors during treatment response is of great importance for immunomodulatory therapies of HCV.

### Proposed therapeutic interventions: Effect of perturbations by inhibition of specific targets

In the context of PN, we had a liberty to showcase various types of inhibitions and knockout experiments. therefore, we studied various host immune regulatory mechanisms which effect the proliferation and survival of immune cells (CD8+ and CD4+ T cells, CTLs, Tregs, Exhausted T cells, and NK cells). Such regulatory mechanisms are necessary in maintaining the normal physiology and to help maintain a balance amongst immune related responses by attenuating them and limit the injury to the tissue due to increased inflammation^24,34,62–64^. As discussed earlier, in the context of viral infections, specifically the HCV, similar mechanisms are activated to help the survival and propagation of the virus^58,64,65^. Based on these facts, it is assumed that HCV regulates hepatic adaptive immunity by activating such regulatory mechanisms. These mechanisms are correspondingly potential therapeutic targets, in order to recover the host immune responses. The critical factors selected during the study of *treatment response model* (IL-12, IL-21, IL-10, exhausted T cells) were perturbated in further *in silico* experiments by increasing or decreasing the levels of various cytokines in the model. IL-12 was not included in further analysis as literature shows that IL-12 is not an effective treatment option for HCV^66^. The simulation analysis enabled us to propose three therapeutic options which might be helpful in immunomodulation during HCV infection treatment along with classical therapy.

### Reversal of T cells exhaustion

**Figure 9:**
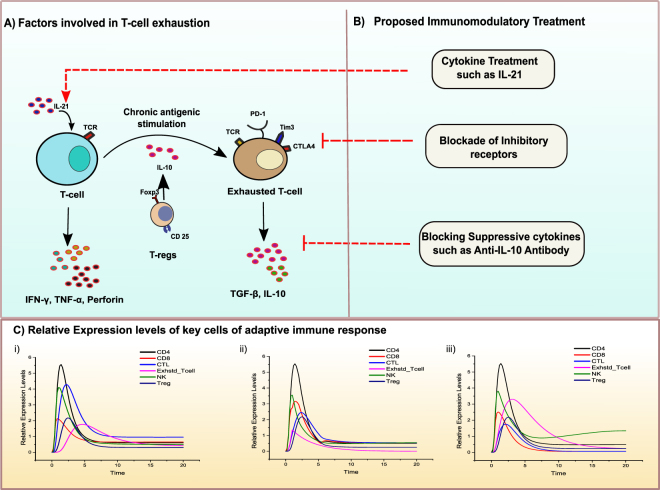
Proposed immunomodulatory treatments and their effect on key cellular immune responses. (**A**) Factors involved in T-cell exhaustion. (**B**) Proposed treatment possibilities and perturbations introduced in the model. (**C**) Relative expression levels of key cellular responses of adaptive immunity including CD4+, CD8+, CTLs, Exhausted T cells, NK, and Tregs. Time units are presented on X-axis while relative expression levels are presented on y-axis represents. (i) The relative expression levels of key cells after IL-21 cytokine treatment. (ii) Relative expression levels of key cells after blocking of exhausted T cells inhibitory receptors. (iii) Relative expression levels of key cells after anti-IL-10 antibody treatment.

The exhausted HCV-specific T cells displaying anergy are present in chronic HCV infection. This could be exploited through a possible therapeutic approach which could effectively reverse the T-cell exhaustion and enable the immune cells to control the virus. As T-cell dysfunction/exhaustion is promoted by several immune regulatory mechanisms including but not limited to as presented in the Fig. 9. The proposed therapeutic options are discussed below where the varying levels of CD4+, CD8+, CTLs, Exhausted T cells, NK, and Tregs can be exploited for the proposed treatments. The proposed Immunomodulatory treatments may include IL 21 therapeutics, blocking of inhibitory receptors and introduction of anti-IL-10 antibodies to helps in a sustained adaptive immune response.

### IL-21 therapeutics can partially restore cytolytic activity

As discussed earlier, IL-21 is a crucial factor in CD4+ T-cell response and also helps in NK cells proliferation as well as mediating the crosstalk amongst T-cells and B-cells^41^. IL-21 may exert its controlling function on Tregs while positively contributing to CD8+ T-cell responses^49^. In the first perturbation experiment, a hypothetical recombinant IL-21 was studied as a co-stimulating immunomodulatory agent in HCV treatment. The levels of IL-21 were increased by introducing a continuous source place in the model, such that its effect on various cells involved in HCV clearance could be analysed simultaneously. Analysis of the simulation results (Fig. 9C (i)) revealed that not only CD4+ T cell response is improved (Black line, Fig. 9C (i)) but also there is a marked increase in the CTLs activity (Blue line). This observation reveals that the hypothetical recombinant IL-12 treatment recovers the CD4+ T-cells in the system and also significantly stimulates cytolytic mechanisms. It aids the control of viremia and facilitate HCV clearance. In terms of a co-stimulatory agent, IL-21 appears be a quite effective cytokine, which can sustain T-cells responses and thus has a potential to be considered in combination therapy to augment the current therapies in controlling the viral infection^49,67,68^.

### Blocking of inhibitory receptors lowers exhausted T-cells count

As discussed earlier the loss of CD4+ T cell help in proliferation of CD8+ T-cells results in CD8+ T cell exhaustion and persistent. Helper CD4+ T cells are significant in the activation of DCs to prime CD8+ T cells. However, the sustained up-regulation of inhibitory receptors PD-1, CTLA4 and Tim3. The HCV core protein strongly upregulates the expression of PD-L1, a ligand for PD-1 receptor, which is known to promote T cell dysfunction^69^. Blocking these inhibitory receptor poses a significant therapeutic option as several studies on various viruses have shown a significant degree of therapeutic promise^70^. Subsequently, in the next perturbation experiment, we blocked the T cells expressing inhibitory receptors to measure the effects on cellular responses. PD-1 blockage does not seem clinically wise decision based on its importance in maintaining normal physiology of the liver. Thus blockade of Tim-3^71^ or CTLA-4, may hold some immunotherapeutic promise. Upon inhibition of cells expressing the inhibitory receptors a considerable reduction in the viremia along with expression of Th1 profile is observed. From Fig. 9C (ii), it can be observed that the exhausted T cells are comparatively low in proportion (Pink line) and the CD8+ T cell (red line) are seen to be slightly highly expressed.

### Anti-IL-10 antibody helps in a sustained NK cell response

Another plausible immunomodulatory strategy could be counteracting IL-10 to reduce its regulatory effect on T-cell maturation and proliferation. Enhanced production of IL-10 cytokine is noted in earlier studies due the effects of core protein of HCV^72^. As IL-10 is an immunomodulatory cytokine which is primarily considered to reduce the cytotoxic potential of T cells as well as NK cells^58^. However, the immunomodulatory potential of IL-10 is also essential for regulating inflammatory responses and helps to reduce immune related tissue injury^59^. IL-10 also inhibits IL-12 production^65,73^, even though IL-12 helps in the activation of NK cells via DCs. The inhibitory effects of IL-10 on activated macrophages has also evidence by decreasing TNF-α^74^. Furthermore, it is also known that IFN-γ suppresses the IL-10 concentration levels^58,60^. Thus, the ratio of IL-10 to IFN-γ is critical for deciding the fate of infection^6^. The hypothesis saying that if IL-10 production is blocked/inhibited, it will result in the improvement of HCV specific T-cell response was tested further. Hence, we designed an *in-silico* experiment to check the effect of decreased concentrations of IL-10 in the system by introducing anti-IL-10 antibody. Figure 9C (ii) shows the simulation results of the perturbation experiment. Apart from the rescue of HCV-specific CD8+ T cell responses, an increase in CD4+ response is also observed. IL-10 antibody treatment result in a sustained NK cell response which is quite necessary for the upregulation of innate and adaptive immunity. Thus, it seems that the higher percentage of IL-10 in chronic HCV infection represses the NK cell function. This repression can be overcome by anti-IL-10 antibody as shown by the model simulation experiment.

Hence, it is suggested that inhibition of various co-inhibitory pathways via hypothesized inhibitors, IL-21 treatment and IL-10 antibody may differentially enhance CTL effector functions, CD4+ cells and likely to improve a therapeutic response when used in combination to other conventional therapies available such as IFN-α/RBV.

## Discussion

Adaptive immune responses take weeks or sometime months to initiate after established viral infection. The role of liver specific aspects as well as viral proteins in the attenuation of adaptive immune responses during HCV infection is still unclear. Immunomodulatory activity of HCV proteins mediated by envelope protein E2 and core proteins is reinforced by *in vitro* cell culture experiments^46,75^. NK cells are a vital direct mediator of immune responses and it might be suppressed by the HCV E2 protein. Similarly, HCV core-mediated DC dysfunction occurs via attenuating of IL-12. Thus, inhibition of antigen presentation on DCs might be interceded further by the effects of viral proteins. Furthermore, T-cell exhaustion is critical determinant in chronic infection. Similarly, it is observed that those patients who can clear the infection show mature CD8+ memory cells maintained by increased number of HCV specific CD8+ cells. Failure to clear the infection results in the persistent display of HCV peptides on the hepatocyte surface, Immune mediate liver injury is the consequence of chronic activation or presence of CTLs. Additionally, the increased production of TGF-β and other related pro-inflammatory cytokines activates stellate cells, which are the primary cause of fibrosis, which results in significant liver damage. A strong antibody response to HCV infection is detected quite early in the infection phase. However, the functional capability of the neutralizing antibodies is quite low. Clearance of infection require vigorous, robust and multi-specific antiviral host immune response^76^.

However, the fact that despite such an adverse scenario, a considerable percentage of individuals clear the virus without any treatment, termed as *spontaneous clearance*. This phenomenon offers hope that somehow fine-tuning the system towards T cells may provide a plausible direction for the resolution of viral infection in the infected individuals. The treatment options available for now are classical IFN-α/RBV immunomodulatory therapy and recently introduced DAAs which work by blocking specific viral proteins. Immunomodulatory therapy of HCV involving IFN-α is still considered SOC therapy for most of the patients. The introduction of direct acting antivirals (DAAs) have significantly increased the treatment outcomes but it is important to note that these treatments have associated limitations. Resistance, toxicity and pre-mature cessation of therapy is a major concern for these targeted therapies. This suggests that the host immune response is essential element of therapy for HCV elimination. Thus, immune modulation might prove to be an effective regimen when considering combination therapies. The strongest evidence that immune modulation is a key component of DAA-based therapies is the difficulty to remove ribavirin from the treatment regimen^77^. Also, it is important to note that it is not enough to stop RNA replication (the goal of DAAs). Eradication of virus completely from the host is also quite significant in maintaining SVR and preventing relapse. Intrinsic immune signatures of the individual host may also determine the outcome of treatment. That is why it is quite significant to overcome immune failure in order to clear the virus from the host body. Also, those patients who have already not responded to IFN-α/RBV therapy, need new therapies which are a long way off. Although both treatments have resulted in good response to therapy in various individuals but still large proportion of patients are null responders and many patients suffer from toxicity and negative side effects of these treatments. Consequently, a need for new therapies and treatments are still quite challenging task ahead. In this regard, new immune modulatory agents which can tweak the system towards more specific T-cell responses with Th1 profile are need of the hour. However, limitation of wet-lab studies does not have the liberty to study all the related immune parameters in single experiment. Similarly, the high rate of data being generated by various individual studies also needs to be analysed as a single system.

In this regard, we have successfully applied PN approach to model a biological signalling network which can be used to study various dynamics of the system in the presence of internal or external stimulus. Most mathematical/computational models require detailed parameters describing the kinetic characteristics of the network, which are typically quite difficult to obtain for all the entities present in the highly interconnected signalling network of proteins. Instead, method used in this study does not necessarily require the detailed quantitative data rather it models signal flow in the PN by token accumulation and dissipation within places (proteins) over time. The tokenized activity-levels computed by this kind of method are abstract quantities whose changes over time correlate to changes that occur in the relative quantities of active proteins present in the cell. Furthermore, it can be assumed that the *in silico* experiments performed, compared the changing levels of proteins relative to the “control”. Moreover as several researchers have observed that the connectivity of a biological network commands, to a great degree, the network’s dynamics^15,78,79^. Many have postulated that biological network connectivity has evolved to have a stabilizing effect on the overall network dynamics, making the network more robust to local fluctuations. Thus, quantitative data such as network parameters, kinetic rates and protein binding affinities are not necessarily required to qualitatively model the network. After verifying and validating the models with reference published data, we utilized the predictive ability of the model to narrow down and tested various immunomodulatory agents in combination with IFN-α/RBV. IL-10 antibody, IL-21 treatment and blocking of inhibitory receptors of T cells which produced promising results in terms of improving CD8+ and CD4+ T cells responses including the reversal of exhausted T cells, increased cytotoxic potential of CTLs and NK cell response. Thus, in our opinion current treatments should be used in conjunction with immunomodulatory agents which can remove the repressive effects of those cytokines responsible for failure of the adaptive responses. IL-10 antibody therapy showed promising results via PN model analysis, the finding postulate that it might be able to better use in prognosis of disease, leading towards reduction in chronicity. This observation is in close agreement with experiments carried out on HBV by Brooks *et al*.^80^. Although IL-10 helps to decrease the disease activity by reducing inflammation mediated tissue damage^81,82^. It is also known from some instances that IL-10 inhibits CD8+ priming and cytolytic mechanisms in HCV infection^83^. Nevertheless, the blockade of IL-10 via antibody therapy showed promising simulation results (Fig. 9). Also, IL-21 perturbation experiment demonstrated decent outcomes in terms of NK cells, CD4+ T cells and CTL responses. IL-21 has been previously shown to regulate effector function of CD8+ T cells^49^. The constructed therapeutic PN model also highlighted the importance of IL-21 cytokine in relation to improvement in treatment response.

Thus, in our opinion these treatment options should be considered for combination therapy regimens including other direct acting antivirals and immunomodulatory agents. hence, HCV replication could be better controlled simultaneously DAA along with improving immune responses. These and other potential combinations can be widely tested through our PN models and the best outcome can be subjected to *in vitro* experimentations. We believe that these approaches will contribute immensely to complement other methods in the biological predictions regarding immune control of other infections as well.

## Methodology

**Figure 10:**
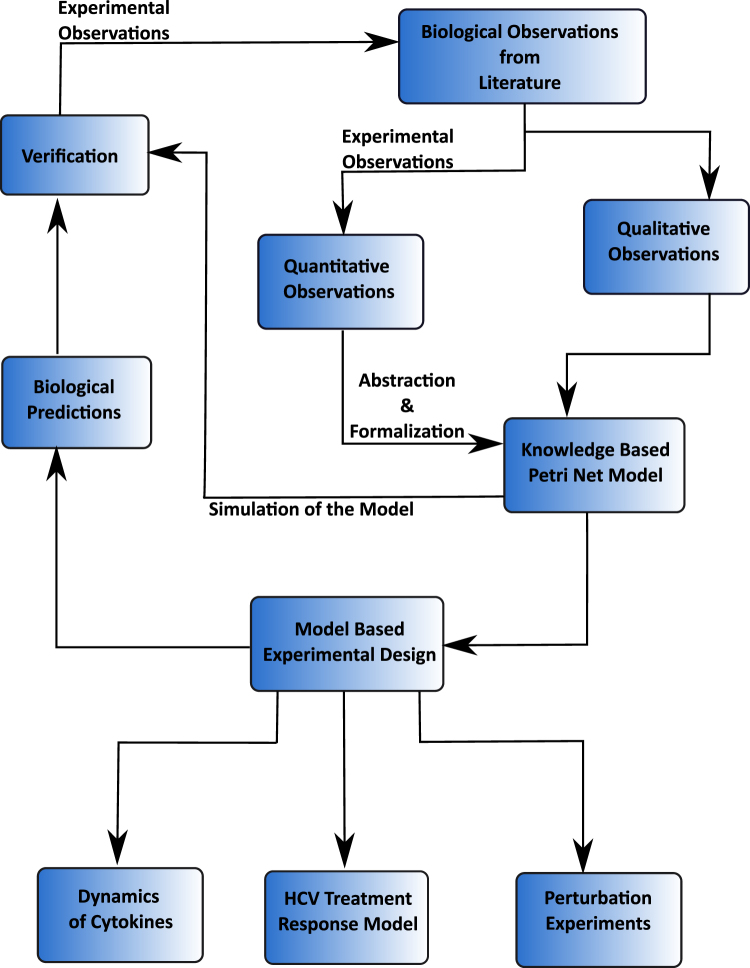
The flowchart represents basic framework of methodology followed to model HCV-induced immune responses using Petri Nets (PN).

The modelling approach employed in this study is demonstrated in Fig. 10. The approach is adapted from our previous pilot study^32^ to systematically build an adaptive immune signalling PN model.

### Overview of the modelling approach

In-depth literature survey of the experimental studies facilitated in generating a logic-based diagram of a comprehensive adaptive immune signalling pathways in response to HCV infection. It signifies imperative signalling pathways triggered during HCV infection in the form of a comprehensive integrated network. This constructed logical network was then subjected to PN modelling, a mathematical formalism for network construction, analysis, and simulation^13,14,84^. After model generation, various dynamic behaviours were studied via simulations run to check for uniformity and agreement with the published data. The modelling framework efficiently represent the constructed immune signalling network along with computing the eminence of various components and processes occurring within the network. The basic model was then extended to perform some *in silico* experiments to predict various outcomes under altering inducements.

### Conversion of the pathway into computable format

PN model of integrated signalling pathway for adaptive immune responses against HCV was constructed employing continuous Petri net approach using tool Snoopy 2.0^85^. It was a step-wise process to build the *baseline model* and later extending it to include other related parameters of treatment response. The model was then checked and verified for correctness, completeness and consistency according to the PN theory.

### Continuous Petri net (CPN)

CPN is an extension of PN^84^ in which the marking is given by positive real number represented by tokens. The value of token represents concentration. The semantic of a continuous PN is given by the corresponding set of ordinary differential equations (ODEs), describing the continuous change over time on the token value of a given place. Where the pre-transition flow results in a continuous increase and post-transition flow results in a continuous decrease. CPNs are presented as a single holistic system to analyse the biological behaviour of each entity especially for *in silico* experiments. Biological regulatory networks, physiochemical networks, gene regulation, transcriptional, epigenetic, protein-protein interactions and signal transduction can be easily modelled using CPN^86^.

In a typical PN model, circles represent places and boxes represent transitions which are continuous in nature, depicting their true nature within complex biological processes. Transitions in PN represent interactions among proteins exhibiting the effects of a source entity on a target entity. Transition firing triggers the source place to release the assigned tokens, called as the *token-count*, which as a result influences the target place. Flow of token in such a way enables the signal propagation through directed interactions within a cellular signalling pathway. This token flow also depends upon the rates of transitions which corresponds to relative concentration levels of reactants and thus can be used to model biological interactions and related enzyme kinetics. PNs can easily create models having all continuous transitions, whose rates are differential equations depending on the place markings or tokens (represented by dots or numbers within the places). The constructed PN models include places (representing genes/proteins) and transitions (representing processes such as activation, inactivation) connected via arcs (edges). Inhibitory arcs are also supported in CPNs where an inhibitory arc inhibits the token flow from either an input place to a transition or from a place to the transition. This feature is very useful to model gene/protein repression in regulatory networks and to perform various *in silico* knockout/inhibitory experiments, network dynamics of the designed model were obtained by executing the simulation run for each model. The transitions are fired randomly to simulate the signalling rates through random interaction occurrences. The averaged token-counts are comparable with experimentally measured variations in the relative expression concentrations of distinct entities in the signalling pathway, which reduces the need of kinetic parameters^11^. Thus, the exact kinetic parameters for each enzymatic reaction are not used in the model rather, the relative activity change is determined by simulations run. The model is based on the assumption that the main protagonist in the signal propagation through a network are the connections amongst various entities involved^15^. These connections (positive, negative) determine the effector functions of the signalling network. The model uses this interconnectivity of the entities and forms a dynamic system which evolves which time.

Formal definition of Petri nets are as follows:

#### Directed Bipartite Graph

A directed bipartite graph is a special case in graph theory having two distinct subsets of vertices in such a way that subsets do not have any common elements, and edges always link the members from different subsets.

**Definition 1** (**Standard Petri net**):

“*A standard Petri net is a quadruple N = (P, T, f, m0)*,

*where:*

*P, T are finite, non-empty, disjoint sets*. $P=\{{P}_{1},\,{P}_{2},\,{P}_{3},\ldots \ldots {P}_{n}\}$ *is the set of places and* $T=\{{T}_{1},\,{T}_{2},\,{T}_{3},\,\ldots \ldots ,\,{T}_{m}\}$ *is the set of transitions*.

$f:((P\times T)\cup (\,T\,\times P))\to {{\mathbb{Z}}}_{\ge 0}$*defines the set of directed arcs, weighted by non-negative integer values*.

${m}_{0}:P\to {{\mathbb{Z}}}_{\ge 0}$ *gives the initial marking* ^87^ *”. (Section 2.2, Page number 6)*

**Definition 2** (**Continuous Petri net**):

“*A continuous Petri net is a quintuple CPN* = *(P, T, f, v, m0), where:*

*P, T are finite, non-empty, disjoint sets. P is the set of continuous places. T is the set of continuous transitions*.

$f:((P\times T)\cup (T\times P))\to {{\mathbb{R}}}_{0\,}^{+}$ *defines the set of directed arcs, weighted by non-negative integer values*

$v:T\to H$ *is a function, which assigns a firing rate function h*_*t*_ *to each transition t, whereby*

$H\,:\,={\cup }_{t\in T}\{{h}_{t}|{h}_{t}:{{\mathbb{R}}}^{|\cdot t|}\to {{\mathbb{R}}}^{+}\}$ *is the set of all firing rate functions, and* $v(t)={h}_{t}$ *for all transitions* $t\in T$

${m}_{0}:P\to {{\mathbb{R}}}_{0}^{+}$ *gives the initial marking”*. (Section 5.2, Page no.52^87^)

### Verification of the model

The initial verification of the model representing HCV-induced adaptive immune signalling was performed using theoretical assumptions, where token numbers represent the relative expression level of an entity, corresponding to a gene inhibitory/knockout model. Simulation of the system is carried out using the PN algorithm in which the initial marking is either 0 (null expression) or 100 (expressed gene) for entry nodes (HCV). It demonstrated that the model suitably imitates the observations derived from multiple experimental analysis. In order to verify and validate our generated models, appropriate simulations were used to measure the key parameters of immune function which presented an integrated view of the entities involved in acute, chronic and resolved HCV infection under stress of various internal and external stimuli. Comparing the results of these simulations with the inferences from published data led to the verification of the model. Subsequently, perturbations were introduced in the model to get insights into the trends in molecular activity in response to exterior stimuli such as therapy (IFN-α/RBV) or immune modulation. In this study, we only analysed the qualitative aspect of data and behaviours and not the absolute quantitative values, to present the system in a convenient and manageable way and to provide simplifications over the *in vivo* conditions to study the phenomenon of cytokine dynamics and treatment responses against HCV infection.

## Conclusion

In addition to expanding the knowledge of signalling pathways of the immune system, a major challenge for the future studies is to pinpoint exact functions of the entities involved in these pathways in the context of infectious diseases. The role of several cytokines in varying conditions and/or in conjunction with other inflammatory responses are determining factors of the outcome of infection, and hence critical in understanding the dynamics of specific immune responses. This will also be very essential for progressing towards the design of an effective vaccine against HCV. As one of major challenges in this area has been the lack of understanding of the requirements for the induction of protective immunity. Though, challenging but we were able to construct a comprehensive model of the adaptive immune system provoked in responses to HCV infection. The model provided significant insights into the immune dynamics in response to viral infections as well as effective perturbation experiments. We strongly believe that similar prior knowledge based modelling approach could be better exploited to test several hypotheses and would poses a great benefit in terms of analysis time, cost and labour. Such model-based studies can be extended to other infections systems to test specific hypotheses and introduce a real-time interventions experiments, the best scenarios could later be verified and confirmed for promising outcomes through *in vitro/in vivo* experiments.

## Electronic supplementary material

Supplementary File 1

## References

[1] Blach S (2017). Global prevalence and genotype distribution of hepatitis C virus infection in 2015: a modelling study. The Lancet Gastroenterology & Hepatology.

[2] Thimme R, Binder M, Bartenschlager R (2012). Failure of innate and adaptive immune responses in controlling hepatitis C virus infection. FEMS microbiology reviews.

[3] Heim MH, Thimme R (2014). Innate and adaptive immune responses in HCV infections. Journal of hepatology.

[4] Horner SM, Gale M (2013). Regulation of hepatic innate immunity by hepatitis C virus. Nature medicine.

[5] Bowen DG, Walker CM (2005). Adaptive immune responses in acute and chronic hepatitis C virus infection. Nature.

[6] Fallahi, P. *et al*. Cytokines and HCV-related disorders. *Clinical and Developmental Immunology* **2012** (2012). 10.1155/2012/468107 PMC3352261 22611419

[7] Rosen HR (2013). Emerging concepts in immunity to hepatitis C virus infection. The Journal of clinical investigation.

[8] Klipp, E., Liebermeister, W., Wierling, C., Kowald, A. & Herwig, R. *Systems biology: a textbook*. (John Wiley & Sons, 2016).

[9] Palsson, B. & Palsson, B. Ø. *Systems biology*. (Cambridge university press, 2015).

[10] Motta S, Pappalardo F (2013). Mathematical modeling of biological systems. Briefings in Bioinformatics.

[11] Polak ME, Ung CY, Masapust J, Freeman TC, Ardern-Jones MR (2017). Petri Net computational modelling of Langerhans cell Interferon Regulatory Factor Network predicts their role in T cell activation. Scientific Reports.

[12] Le Novère N (2015). Quantitative and logic modelling of molecular and gene networks. Nature Reviews Genetics.

[13] Sackmann A, Heiner M, Koch I (2006). Application of Petri net based analysis techniques to signal transduction pathways. BMC bioinformatics.

[14] Chaouiya C (2007). Petri net modelling of biological networks. Briefings in Bioinformatics.

[15] Ruths D, Muller M, Tseng J-T, Nakhleh L, Ram PT (2008). The Signaling Petri Net-Based Simulator: A Non-Parametric Strategy for Characterizing the Dynamics of Cell-Specific Signaling Networks. PLoS Computational Biology.

[16] Rehermann B (2009). Hepatitis C virus versus innate and adaptive immune responses: a tale of coevolution and coexistence. The Journal of clinical investigation.

[17] Gorham, J. D. In *Liver Immunology* 61–70 (Springer, 2007).

[18] Takahashi K (2010). Plasmacytoid dendritic cells sense hepatitis Cvirus–infected cells, produce interferon, and inhibit infection. Proceedings of the National Academy of Sciences.

[19] Fan Z, Huang X-L, Kalinski P, Young S, Rinaldo CR (2007). Dendritic cell function during chronic hepatitis C virus and human immunodeficiency virus type 1 infection. Clinical and Vaccine Immunology.

[20] Vivier E (2011). Innate or adaptive immunity? The example of natural killer cells. science.

[21] Nellore A, Fishman JA (2011). NK cells, innate immunity and hepatitis C infection after liver transplantation. Clinical Infectious Diseases.

[22] Rehermann B (2015). Natural killer cells in viral hepatitis. CMGH Cellular and Molecular Gastroenterology and Hepatology.

[23] Amadei B (2010). Activation of natural killer cells during acute infection with hepatitis C virus. Gastroenterology.

[24] Belkaid Y, Rouse BT (2005). Natural regulatory T cells in infectious disease. Nature immunology.

[25] Cook, K. D., Waggoner, S. N. & Whitmire, J. K. NK cells and their ability to modulate T cells during virus infections. *Critical Reviews™ in Immunology* **34** (2014). 10.1615/critrevimmunol.2014010604 PMC4266186 25404045

[26] Yoon JC, Yang CM, Song Y, Lee JM (2016). Natural killer cells in hepatitis C: Current progress. World journal of gastroenterology.

[27] Neumann-Haefelin, C. & Thimme, R. In *Hepatitis C Virus: From Molecular Virology to Antiviral Therapy* 243–262 (Springer, 2013).

[28] Eckels DD, Wang H, Bian TH, Tabatabai N, Gill JC (2000). Immunobiology of hepatitis C virus (HCV) infection: the role of CD4 T cells in HCV infection. Immunological reviews.

[29] Shin E-C, Sung PS, Park S-H (2016). Immune responses and immunopathology in acute and chronic viral hepatitis. Nature Reviews Immunology.

[30] Veiga-Parga T, Sehrawat S, Rouse BT (2013). Role of regulatory T cells during virus infection. Immunological reviews.

[31] Zhang P (2009). Depletion of interfering antibodies in chronic hepatitis C patients and vaccinated chimpanzees reveals broad cross-genotype neutralizing activity. Proceedings of the National Academy of Sciences.

[32] Obaid A (2015). Modeling and analysis of innate immune responses induced by the host cells against hepatitis C virus infection. Integrative Biology.

[33] Alhetheel A (2016). Assessment of pro-inflammatory cytokines in sera of patients with hepatitis C virus infection before and after anti-viral therapy. The Journal of Infection in Developing Countries.

[34] Cacciarelli TV, Martinez OM, Gish RG, Villanueva JC, Krams SM (1996). Immunoregulatory cytokines in chronic hepatitis C virus infection: Pre-and posttreatment with interferon alfa. Hepatology.

[35] Day CL (2002). Broad specificity of virus-specific CD4+ T-helper-cell responses in resolved hepatitis C virus infection. Journal of virology.

[36] Smyk-Pearson S (2008). Spontaneous recovery in acute human hepatitis C virus infection: functional T-cell thresholds and relative importance of CD4 help. Journal of virology.

[37] Marras, F., Bozzano, F., Ascierto, M. L. & De Maria, A. Baseline and dynamic expression of activating NK cell receptors in the control of chronic viral infections: the paradigm of HIV-1 and HCV. *Frontiers in immunology* **5** (2014). 10.3389/fimmu.2014.00305 PMC4078246 25071766

[38] Larkin J, Bost A, Glass JI, Tan S-L (2006). Cytokine-activated natural killer cells exert direct killing of hepatoma cells harboring hepatitis C virus replicons. Journal of interferon & cytokine research.

[39] Frese M (2002). Interferon-γ inhibits replication of subgenomic and genomic hepatitis C virus RNAs. Hepatology.

[40] Zhao J, Zhao J, Perlman S (2012). Differential effects of IL-12 on Tregs and non-Treg T cells: roles of IFN-γ, IL-2 and IL-2R. PloS one.

[41] Parrish-Novak J, Dillon SR, Nelson A, Hammond A (2000). Interleukin 21 and its receptor are involved in NK cell expansion and regulation of lymphocyte function. Nature.

[42] Pène J (2004). Cutting edge: IL-21 is a switch factor for the production of IgG1 and IgG3 by human B cells. The Journal of Immunology.

[43] Radziewicz H (2007). Liver-infiltrating lymphocytes in chronic human hepatitis C virus infection display an exhausted phenotype with high levels of PD-1 and low levels of CD127 expression. Journal of virology.

[44] Rutebemberwa A (2008). High-programmed death-1 levels on hepatitis C virus-specific T cells during acute infection are associated with viral persistence and require preservation of cognate antigen during chronic infection. The Journal of Immunology.

[45] Cho H, Kang H, Lee HH, Kim CW (2017). Programmed Cell Death 1 (PD-1) and Cytotoxic T Lymphocyte-Associated Antigen 4 (CTLA-4) in Viral Hepatitis. International Journal of Molecular Sciences.

[46] Tseng C-TK, Klimpel GR (2002). Binding of the hepatitis C virus envelope protein E2 to CD81 inhibits natural killer cell functions. Journal of Experimental Medicine.

[47] Pei-Lin Cheng M-HC, Chaol C-H, Lee Y-HW (2004). Hepatitis C viral proteins interact with Smad3 and differentially regulateTGF-b/Smad3-mediated transcriptional activation. Oncogene.

[48] Tran DQ (2011). TGF-β: the sword, the wand, and the shield of FOXP3+ regulatory T cells. Journal of molecular cell biology.

[49] Feng G (2013). HCV-specific interleukin-21+ CD4+ T cells responses associated with viral control through the modulation of HCV-specific CD8+ T cells function in chronic hepatitis C patients. Molecules and cells.

[50] Lawitz E (2014). Simeprevir plus sofosbuvir, with or without ribavirin, to treat chronic infection with hepatitis C virus genotype 1 in non-responders to pegylated interferon and ribavirin and treatment-naive patients: the COSMOS randomised study. The Lancet.

[51] Stevenson NJ (2011). Ribavirin enhances IFN-α signalling and MxA expression: a novel immune modulation mechanism during treatment of HCV. PloS one.

[52] Testoni, B., Levrero, M. & Durantel, D. Mechanism of action of ribavirin in anti-HCV regimens: new insights for an age-old question? *Gut*, gutjnl-2013-304528 (2013). 10.1136/gutjnl-2013-304528 23661602

[53] Zhou S, Liu R, Baroudy BM, Malcolm BA, Reyes GR (2003). The effect of ribavirin and IMPDH inhibitors on hepatitis C virus subgenomic replicon RNA. Virology.

[54] Werner JM (2014). Ribavirin improves the IFN-γ response of natural killer cells to IFN-based therapy of hepatitis C virus infection. Hepatology.

[55] Yoneda S (2011). Association of serum cytokine levels with treatment response to pegylated interferon and ribavirin therapy in genotype 1 chronic hepatitis C patients. Journal of Infectious Diseases.

[56] Kamal SM, Fehr J, Roesler B, Peters T, Rasenack JW (2002). Peginterferon alone or with ribavirin enhances HCV-specific CD4+ T-helper 1 responses in patients with chronic hepatitis C. Gastroenterology.

[57] Nakamura I (2015). Restoration of natural killer cell activity by pegylated interferon-alpha/ribavirin therapy in chronic hepatitis C patient. Hepatology Research.

[58] Blackburn SD, Wherry EJ (2007). IL-10, T cell exhaustion and viral persistence. Trends in microbiology.

[59] Fiorentino DF, Zlotnik A, Mosmann T, Howard M, O’garra A (1991). IL-10 inhibits cytokine production by activated macrophages. The Journal of Immunology.

[60] Hu X (2006). IFN-γ suppresses IL-10 production and synergizes with TLR2 by regulating GSK3 and CREB/AP-1 proteins. Immunity.

[61] Pirisi M (1997). Endogenous interferon-α concentration and outcome of interferon treatment in patients with chronic hepatitis C. Digestive diseases and sciences.

[62] Barth H (2003). Analysis of the effect of IL-12 therapy on immunoregulatory T-cell subsets in patients with chronic hepatitis C infection. Hepato-gastroenterology.

[63] Boer, M. C., Joosten, S. A. & Ottenhoff, T. H. Regulatory T-cells at the interface between human host and pathogens in infectious diseases and vaccination. *Frontiers in immunology* **6** (2015). 10.3389/fimmu.2015.00217 PMC4426762 26029205

[64] Cabrera R (2004). An immunomodulatory role for CD4+ CD25+ regulatory T lymphocytes in hepatitis C virus infection. Hepatology.

[65] Aste-Amezaga M, Ma X, Sartori A, Trinchieri G (1998). Molecular mechanisms of the induction of IL-12 and its inhibition by IL-10. The Journal of Immunology.

[66] Pockros PJ (2003). A multicenter study of recombinant human interleukin 12 for the treatment of chronic hepatitis C virus infection in patients nonresponsive to previous therapy. Hepatology.

[67] MacParland SA (2016). HCV specific IL-21 producing T cells but not IL-17A producing T cells are associated with HCV viral control in HIV/HCV coinfection. PloS one.

[68] John SY, Du M, Zajac AJ (2009). A vital role for interleukin-21 in the control of a chronic viral infection. science.

[69] Yao ZQ, King E, Prayther D, Yin D, Moorman J (2007). T cell dysfunction by hepatitis C virus core protein involves PD-1/PDL-1 signaling. Viral immunology.

[70] Salem ML, El-Badawy A (2015). Programmed death-1/programmed death-L1 signaling pathway and its blockade in hepatitis C virus immunotherapy. World journal of hepatology.

[71] Golden-Mason L (2009). Negative immune regulator Tim-3 is overexpressed on T cells in hepatitis C virus infection and its blockade rescues dysfunctional CD4+ and CD8+ T cells. Journal of virology.

[72] Fernandez-Ponce C, Dominguez-Villar M, Aguado E, Garcia-Cozar F (2014). CD4+ primary T cells expressing HCV-core protein upregulate Foxp3 and IL-10, suppressing CD4 and CD8 T cells. PloS one.

[73] Rahim SS, Khan N, Boddupalli CS, Hasnain SE, Mukhopadhyay S (2005). Interleukin-10 (IL-10) mediated suppression of IL-12 production in RAW 264.7 cells also involves c-rel transcription factor. Immunology.

[74] O’Farrell AM, Liu Y, Moore KW, Mui ALF (1998). IL-10 inhibits macrophage activation and proliferation by distinct signaling mechanisms: evidence for Stat3-dependent and-independent pathways. The EMBO journal.

[75] Waggoner SN, Hall CH, Hahn YS (2007). HCV core protein interaction with gC1q receptor inhibits Th1 differentiation of CD4+ T cells via suppression of dendritic cell IL-12 production. Journal of leukocyte biology.

[76] Lauer, G. M. & Kim, A. Y. (The University of Chicago Press, 2006).

[77] Ahlén G (2013). Containing “The Great Houdini” of viruses: combining direct acting antivirals with the host immune response for the treatment of chronic hepatitis C. Drug Resistance Updates.

[78] Li C (2006). Structural modeling and analysis of signaling pathways based on Petri nets. Journal of bioinformatics and computational biology.

[79] Klemm K, Bornholdt S (2005). Topology of biological networks and reliability of information processing. Proceedings of the National Academy of Sciences of the United States of America.

[80] Brooks DG (2006). Interleukin-10 determines viral clearance or persistence *in vivo*. Nature medicine.

[81] Moore KW, de Waal Malefyt R, Coffman RL, O’Garra A (2001). Interleukin-10 and the interleukin-10 receptor. Annual review of immunology.

[82] Nelson DR, Lauwers GY, Lau JY, Davis GL (2000). Interleukin 10 treatment reduces fibrosis in patients with chronic hepatitis C: a pilot trial of interferon nonresponders. Gastroenterology.

[83] Niesen E, Schmidt J, Flecken T, Thimme R (2014). Suppressive Effect of Interleukin 10 on Priming of Naive Hepatitis C Virus–Specific CD8+ T Cells. The Journal of infectious diseases.

[84] David, R. & Alla, H. *Discrete, continuous, and hybrid Petri nets*. (Springer Science & Business Media, 2010).

[85] Heiner, M., Herajy, M., Liu, F., Rohr, C. & Schwarick, M. Snoopy–a unifying Petri net tool. *Application and Theory of Petri Nets*, 398–407 (2012).

[86] Matsuno, H., Doi, A., Nagasaki, M. & Miyano, S. In *Pacific Symposium on Biocomputing*. 87 (World Scientific Press Singapore).

[87] Blätke, M. A., Heiner, M. & Marwan, W. Petri Nets in Systems Biology. (Technical Report, Otto-von-Guericke University Magdeburg, 2011).

[88] Herzer K (2003). Upregulation of major histocompatibility complex class I on liver cells by hepatitis C virus core protein via p53 and TAP1 impairs natural killer cell cytotoxicity. Journal of virology.

