# Supplementary material for: Model of the adaptive immune response system against HCV infection reveals potential immunomodulatory agents for combination therapy
Source: Sci Rep. 2018 Jun 11;8:8874. doi: 10.1038/s41598-018-27163-0 (PMC5995896; doi:10.1038/s41598-018-27163-0)
Supplement: Supplementary file 1 [file 41598_2018_27163_MOESM1_ESM.pdf]

Model of the adaptive immune response system against HCV infection reveals potential immunomodulatory agents for combination therapy

Ayesha Obaid[1], Anam Naz[1], Aqsa Ikram[1], Faryal Mehwish Awan[1], Abida Raza[2], Jamil Ahmad[3], Amjad Ali[1*]

[1]Atta-ur-Rahman School of Applied Biosciences (ASAB), National University of Sciences and Technology (NUST), Islamabad, Pakistan

[2]National Institute of Lasers and Optronics (NILOP), Islamabad, Pakistan

[3]Research Center for Modeling and Simulation (RCMS), National University of Sciences and Technology (NUST), Islamabad, Pakistan

*Corresponding Author

Email: amjaduni@gmail.com

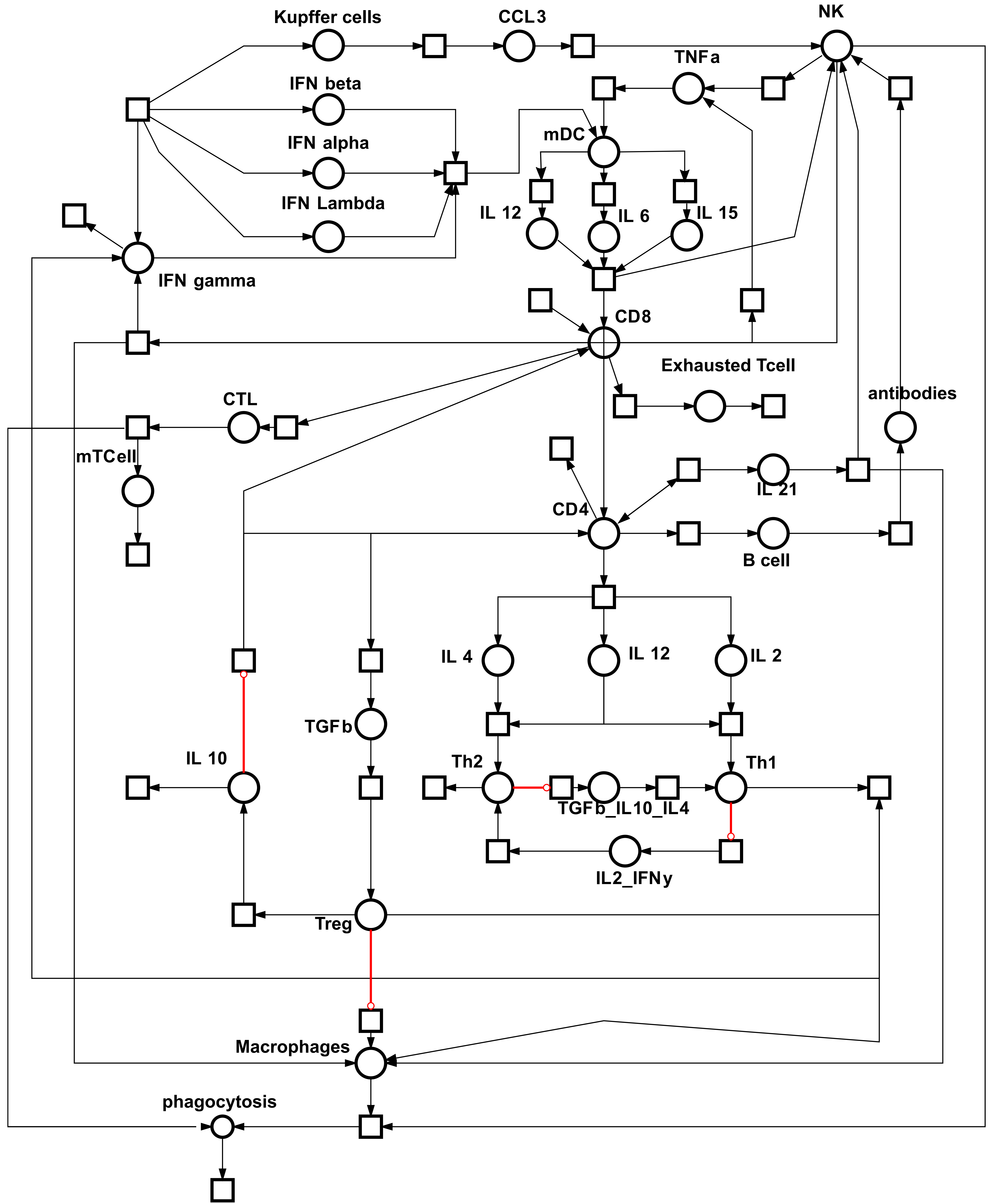
Kupffer cells
CCL3
NK
TNFa
IFN beta
IFN alpha
IFN Lambda
mDC
IL 12
IL 6
IL 15
IFN gamma
CD8
Exhausted Tcell
CTL
mTCell
antibodies
IL 21
CD4
B cell
IL 4
IL 12
IL 2
TGFb
IL 10
Th2
Th1
TGFb_IL10_IL4
IL2_IFNy
Treg
Macrophages
phagocytosis
